# Subjective Sleep Quality Is Associated with Post-Exercise Appetite Loss in Female University Athletes: An Exploratory Cross-Sectional Study

**DOI:** 10.3390/sports14040157

**Published:** 2026-04-16

**Authors:** Shizuka Murano, Yoko Amano, Tomoko Kaburagi

**Affiliations:** 1 Graduate School of Sports and Health Science, Daito Bunka University, Saitama 355-8501, Japan; s22281006@st.daito.ac.jp; 2 Division of Nutrition, Department of Health Science, Daito Bunka University, Saitama 355-8501, Japan; satou@ic.daito.ac.jp

**Keywords:** post-exercise appetite loss, sleep quality, female university athletes, lifestyle-related factors

## Abstract

Post-exercise appetite loss may interfere with adequate recovery nutrition in athletes; however, the substantial inter-individual variability in appetite responses remains insufficiently understood. This exploratory cross-sectional study investigated lifestyle- and health-related factors associated with post-exercise appetite loss in 35 female university athletes. Appetite loss was assessed as a self-reported binary outcome (often, sometimes/never). Associations with subjective sleep quality and other lifestyle-related variables were examined using contingency analysis, followed by exploratory logistic regression. Post-exercise appetite loss was reported by 74.3% of participants and did not differ across sports disciplines, indicating that the sport type alone did not explain the observed variability. Poor/fair subjective sleep quality was associated with appetite loss (OR = 11.6, 95% CI: 1.9–73.6) and remained associated in the multivariate model. Other lifestyle-related variables were not independently associated. These findings imply a potential connection linking post-exercise appetite responses in female university athletes to broader lifestyle-related factors, particularly subjective sleep quality, rather than exercise characteristics alone. Monitoring sleep quality may therefore help identify athletes who may be at risk of insufficient post-exercise energy intake and compromised recovery. Further studies with larger samples and longitudinal designs are needed to clarify these relationships.

## 1. Introduction

Adequate energy intake following exercise is essential for recovery, training adaptation, and maintenance of health in athletes [1]. Inadequate post-exercise energy intake may impair muscle repair, immune function, and endocrine balance, thereby compromising daily conditioning and subsequent training performance. University athletes, who must balance intensive training with academic demands and daily life, may be particularly susceptible to suboptimal recovery due to constraints on sleep, eating opportunities, and recovery times. This dual burden often results in irregular daily schedules, sleep restriction, accumulated fatigue, and psychological stress. Female university athletes are particularly vulnerable to insufficient energy intake and low energy availability, which are associated with impaired performance and adverse health outcomes [1].

In recent years, increasing attention has been paid to the dual-career challenges faced by university athletes at the international level [2,3,4]. Previous research has shown that sport–life balance is closely associated with well-being in university student athletes [5]. Furthermore, female university athletes have been reported to exhibit insufficient energy and micronutrient intakes [6,7,8,9], and large-scale surveys of Japanese college athletes have demonstrated that usual energy intakes often fail to meet recommended standards [7,8,9].

In university athletes, physiological responses to training may interact with psychosocial stressors such as academic workload, recovery imbalance, and sleep disturbance. These factors may collectively influence appetite perception and recovery behaviors, suggesting that post-exercise appetite responses may reflect not only acute physiological mechanisms but also broader lifestyle-related strain. Sleep quality has also been associated with dietary intake in Japanese female university athletes [10], highlighting the importance of daily lifestyle factors in shaping nutritional behaviors. Cross-sectional data have further suggested the coexistence of suboptimal diet and sleep behaviors among student athletes [4].

In practice, coaches and sports dietitians frequently observe that some athletes report appetite loss after training, whereas others do not, even under similar training loads. Exercise-induced appetite suppression has been linked to several physiological mechanisms, including alterations in gut hormones, sympathetic nervous system activation, and transient changes in gastrointestinal blood flow [11,12]. However, these physiological mechanisms alone do not fully explain the substantial interindividual variability observed in real training environments. Behavioral and lifestyle-related factors, including psychological stress, sleep disturbance, and recovery imbalance, may also contribute to variability in appetite perception and eating behavior in athletes. Understanding why certain athletes experience post-exercise appetite loss is therefore important for supporting adequate recovery nutrition intake. Compensatory responses in eating behavior after exercise have also been reported [13].

Beyond acute physiological responses, appetite perception may be influenced by habitual lifestyle factors and subjective health conditions. Poor sleep quality and short sleep duration have been shown to alter appetite-regulating hormones and eating behaviors [14]. Psychological stress has been linked to altered eating behaviors through reward-related pathways [15]. Preliminary observations have also indicated that individual factors may be associated with a history of exercise-induced appetite loss [16]. Gastrointestinal symptoms, such as diarrhea or abdominal discomfort, may further reduce the motivation or capacity to consume food after exercise, particularly under conditions of accumulated fatigue.

Despite its practical relevance, few studies have specifically examined the relationship between daily lifestyle-related subjective conditions and post-exercise appetite loss in female university athletes. Clarifying these associations may help develop individualized strategies to facilitate adequate post-exercise energy intake and prevent energy deficiency in at-risk athletes. Indeed, the importance of nutritional intervention has been highlighted to improve the dietary intake and eating habits of female college athletes. Therefore, this exploratory study aimed to investigate lifestyle- and health-related factors associated with post-exercise appetite loss among female university athletes using a questionnaire-based design.

## 2. Materials and Methods

### 2.1. Participants

Female university athletes belonging to basketball, soccer, and speed skating teams at a university in Japan were invited to participate in this study. These sports were selected to reflect differences in the exercise environment (indoor, outdoor, and cold outdoor conditions). All team members who had been with the athletic club for at least one year were invited to participate. A total of 35 athletes (basketball, *n* = 12; soccer, *n* = 12; speed skating, *n* = 11) provided valid responses and were included in the analysis.

### 2.2. Study Design and Data Collection

A cross-sectional, anonymous, web-based questionnaire survey was conducted in April 2023 using the Google Forms application. The survey link was distributed by team coaches through the university’s sports promotion center. Participants completed the questionnaire after providing informed consent electronically.

### 2.3. Questionnaire Items

Participants reported their lifestyle and health status over the previous 3 months using a structured questionnaire consisting of 15 items (Table S1). Several constructs, including sleep quality, stress level, stress tolerance, gastrointestinal symptoms, fatigue, and appetite loss, were assessed using single-item self-report measures. The questionnaire was developed specifically for this exploratory study based on the previous literature and practical observations in university athletes. Because the primary aim of the study was to explore potential lifestyle-related factors associated with post-exercise appetite responses, brief single-item questions were used to reduce respondent burden in the athlete population. Standardized instruments such as comprehensive sleep or eating behavior scales were not used because the survey was designed to capture multiple lifestyle factors within a brief questionnaire format suitable for athletes during the competitive season. However, this approach may limit construct validity, which should be considered when interpreting the findings.

Post-exercise appetite loss was assessed using a single self-reported item asking whether participants experienced a decrease in appetite after exercise (“often,” “sometimes,” or “never”). Sleep duration, subjective sleep quality, dietary habits (meal frequency, meal quantity, and snacking), perceived stress level, perceived stress tolerance, and physical symptoms (abdominal pain, diarrhea, headache, and fatigue) were assessed using categorical response options.

For statistical analyses, post-exercise appetite loss was dichotomized into “present” (“often” or “sometimes”) and “absent” (“never”) to improve statistical stability and facilitate interpretability given the exploratory nature and small sample size of the study. Similarly, subjective sleep quality (“good” vs. “fair/poor”), usual appetite loss, frequency of diarrhea, and frequency of fatigue were recategorized into binary variables for analysis. Sleep duration was categorized as <7 h and ≥7 h per day. Meal frequency was categorized as <3 meals per day and ≥3 meals per day. Usual meal quantity was categorized as sufficient versus insufficient intake. Snacking before exercise was categorized as present versus absent. Perceived stress level was categorized as high versus low. Perceived stress tolerance was categorized as low versus high. Physical symptoms (stomachache, abdominal pain, headache, diarrhea, and fatigue) were categorized as present versus absent.

### 2.4. Statistical Analysis

All statistical analyses were performed using IBM SPSS Statistics for Windows (version 24).

Because this study was designed as an exploratory survey in a specific athlete population, a formal a priori sample size calculation was not performed. Instead, the analysis aimed to identify potential associations that may inform future hypothesis-driven studies with larger samples. Given the exploratory nature of the study and the small sample size, several variables were recategorized to improve statistical stability and interpretability. Post-exercise appetite loss was analyzed as a binary outcome (“present” vs. “absent”). Because only three athletes reported experiencing appetite loss “often,” maintaining three outcome categories would have resulted in sparse cell counts and unstable statistical estimates. Therefore, the variable was dichotomized for contingency analyses and logistic regression modeling. Subjective sleep quality, usual appetite loss, frequency of diarrhea, and frequency of fatigue were also analyzed as binary variables. Although dichotomization may reduce information and potentially attenuate dose–response relationships, this approach was used to improve statistical stability given the limited sample size and sparse cell counts in some categories. Dichotomization improves analytical stability in small samples; it may reduce information and attenuate potential dose–response relationships. Associations between post-exercise appetite loss and lifestyle- and health-related variables were examined using 2 × 2 contingency tables. Fisher’s exact test (two-sided) was used to assess statistical significance. Effect sizes were calculated using the phi (φ) coefficient. Odds ratios (ORs) with 95% confidence intervals (CIs) were computed from contingency tables. In cases of zero-cell counts, ORs were estimated using the Haldane–Anscombe correction (adding 0.5 to each cell) to avoid infinite estimates. To explore factors independently associated with post-exercise appetite loss, an exploratory logistic regression model was subsequently constructed. Multicollinearity among explanatory variables was assessed prior to regression modeling to ensure model stability. Variable selection was based on effect size and statistical significance in contingency table analyses, as well as considerations of model stability given the limited number of non-events. Subjective sleep quality and usual appetite loss were included in the multivariate model. Model fit was assessed using the Hosmer–Lemeshow goodness-of-fit test, and discrimination ability was evaluated using the area under the receiver operating characteristic curve (AUC). Statistical significance was set at *p* < 0.05. Because the study was exploratory, no correction for multiple comparisons was applied.

### 2.5. Ethical Considerations

This study was conducted in accordance with the principles of the Declaration of Helsinki. The study protocol was approved by the Ethics Committee for Human Research at Daito Bunka University (approval number: DHR22-029). Electronic informed consent was obtained from all participants prior to the completion of the survey.

## 3. Results

### 3.1. Prevalence of Post-Exercise Appetite Loss

Among the 35 female university athletes, 26 (74.3%) reported experiencing post-exercise appetite loss. Specifically, 3 athletes reported experiencing appetite loss “often,” 23 reported experiencing it “sometimes,” and 9 (25.7%) reported “never.” Notably, the three athletes reporting “often” were distributed across different sports disciplines (one athlete each from basketball, soccer, and speed skating). Thus, although post-exercise appetite loss was common in this population, a substantial proportion of athletes did not experience appetite suppression, indicating clear inter-individual variability. The prevalence did not significantly differ across sports disciplines (Fisher’s exact test, *p* = 0.881; Table 1), suggesting that factors beyond sport discipline may contribute to this inter-individual variability.

### 3.2. Associations Between Post-Exercise Appetite Loss and Lifestyle- and Health-Related Factors

Associations between post-exercise appetite loss and each predefined lifestyle- and health-related variable were examined using 2 × 2 contingency tables with Fisher’s exact test. Unadjusted odds ratios (ORs) and 95% confidence intervals (CIs) were calculated to estimate the magnitude of associations (Table 2). Subjective sleep quality was significantly associated with post-exercise appetite loss (*p* = 0.006). Athletes reporting fair/poor sleep quality were more likely to report appetite loss than those reporting good sleep quality (φ = 0.50). The unadjusted odds of appetite loss were higher among athletes with fair/poor sleep quality (OR = 11.63, 95% CI: 1.90–73.64). However, the wide confidence intervals indicate limited statistical precision due to the small sample size, and these estimates should therefore be interpreted cautiously. Diarrhea was also associated with post-exercise appetite loss (*p* = 0.015, φ = 0.43). None of the athletes without appetite loss reported diarrhea. Because of zero-cell counts, the odds ratio was estimated using the Haldane–Anscombe correction (OR = 25.61, 95% CI: 1.22–492.9) and should be interpreted cautiously. Due to zero-cell counts, odds ratios were estimated using the Haldane–Anscombe correction (OR = 25.61, 95% CI: 1.22–492.9), and should therefore be interpreted with considerable caution because of the wide confidence interval and sparse data. Usual appetite loss showed a moderate association (φ = 0.29) with an unadjusted OR of 3.77 (95% CI: 0.76–18.73), although this did not reach statistical significance (*p* = 0.119). Fatigue demonstrated a small-to-moderate effect size (φ = 0.23) but was not statistically significant (*p* = 0.178).

Associations for all predefined lifestyle-, stress-, and health-related variables are summarized in Supplementary Table S2. No statistically significant associations were observed for sleep duration, meal frequency, usual meal quantity, snacking before exercise, perceived stress level, perceived stress tolerance, onset of stomachache, abdominal pain, or headache.

### 3.3. Exploratory Logistic Regression Analysis

To examine factors independently associated with post-exercise appetite loss, an exploratory logistic regression model was constructed. Variable selection was based on (1) effect size in contingency table analyses, (2) statistical significance, and (3) model stability considerations given the limited number of non-events (*n* = 9).

Although both sleep quality and diarrhea demonstrated relatively large effect sizes (φ = 0.50 and φ = 0.43, respectively), diarrhea was excluded from the multivariable model due to zero-cell counts and the resulting instability of the odds ratio estimation. Fatigue showed a smaller effect size (φ = 0.23) and did not reach statistical significance. Therefore, subjective sleep quality and usual appetite loss were included in the multivariate analysis. In the multivariate model, fair/poor sleep quality remained independently associated with post-exercise appetite loss (adjusted OR = 9.68, 95% CI: 1.51–61.93, *p* = 0.016, Table 3). In contrast, usual appetite loss was not significantly associated with the outcome after adjustment (adjusted OR = 2.44, 95% CI: 0.40–14.79, *p* = 0.333).

Model fit was acceptable according to the Hosmer–Lemeshow test (χ^2^ = 2.33, *p* = 0.127), and the model demonstrated moderate discrimination (AUC = 0.79). Given the limited sample size, these findings should be interpreted as exploratory.

## 4. Discussion

The present exploratory study suggests that post-exercise appetite loss among female university athletes reflects broader lifestyle- and recovery-related conditions rather than exercise characteristics alone. The absence of sport-discipline differences may suggest that appetite suppression in this population is influenced more by individual recovery imbalance and perceived strain than by discipline-specific training environments. Although appetite suppression following exercise has traditionally been explained by acute physiological mechanisms [11,12], our findings indicate that daily subjective health status—particularly sleep quality and gastrointestinal condition—may be associated with post-exercise appetite responses in this population.

The dual academic and athletic demands characteristic of university athletes may predispose certain individuals to chronic sleep disturbance and recovery imbalance [2,3,4,5]. In the present study, post-exercise appetite loss was not fully explained by exercise-related physiological responses alone; rather, subjective sleep quality emerged as one of the most prominent associated factors in this cohort. This finding suggests that appetite responses after exercise in university athletes may reflect accumulated lifestyle strain and recovery-related conditions. Sleep occupies a central position within this recovery framework. Beyond its well-established role in physical restoration, sleep modulates autonomic balance, hypothalamic appetite-regulating pathways, and reward-related neural circuits that influence food motivation [14,15]. Inadequate or poor-quality sleep may attenuate post-exercise hunger signals, blunt reward sensitivity to food, or increase fatigue-related eating avoidance [17,18]. Repeated sleep restrictions due to early training sessions, academic workload, or irregular daily rhythms may amplify these effects, making sleep quality a practical indicator of recovery status associated with post-exercise nutritional behavior. In the general population, experimental sleep restriction has often been associated with increased appetite through alterations in leptin and ghrelin secretion [14,19]. However, university athletes engaged in intensive training may represent a physiologically distinct population. Many maintain relatively low body fat levels [7] and are exposed to sustained high training loads, resulting in cumulative fatigue and elevated recovery demands. Under such conditions, appetite regulation may not be governed solely by leptin–ghrelin dynamics but also by energy availability status, autonomic strain, and central fatigue. Therefore, it is plausible that sleep–appetite interactions in athletes differ from those observed in non-athletic young adults, and that poor sleep quality may contribute to appetite suppression rather than appetite stimulation following strenuous exercise.

Gastrointestinal symptoms, particularly diarrhea, were also associated with post-exercise appetite loss. Exercise-induced gastrointestinal disturbances are well documented, especially among endurance athletes and during prolonged or high-intensity training [20,21]. Reduced splanchnic blood flow, altered gastrointestinal motility, increased intestinal permeability (“leaky gut”), and mechanical stress may contribute to post-exercise discomfort and altered bowel function [21]. Such disturbances may blunt appetite perception or reduce willingness to eat after exercise. In female university athletes, these physiological stressors may be amplified by accumulated fatigue, psychological strain, irregular meal timing, and limited recovery opportunities. Gastrointestinal discomfort, even when mild, may therefore act as an additional barrier to post-exercise feeding. Although the independent contribution of gastrointestinal symptoms could not be confirmed in the multivariable model due to statistical instability, the observed association suggests that gut-related responses to training load warrant further investigation, particularly in athletes exposed to repeated endurance-based stress.

Interestingly, perceived stress and stress tolerance were not independently associated with post-exercise appetite loss. This does not imply that stress is unimportant; rather, it suggests that its effects may be mediated through downstream pathways such as sleep disturbance, autonomic dysregulation, or gastrointestinal symptoms [15]. In this cohort, sleep quality and gastrointestinal condition may have captured these integrated recovery-related processes more directly. Taken together, these findings support a broader conceptual view in which post-exercise appetite loss is not merely an acute hormonal response [11,12], but a manifestation of accumulated lifestyle strain and recovery imbalance. Recent work has highlighted the interaction between lifestyle stressors, recovery balance, and health-related behaviors among university students, suggesting that behavioral responses such as appetite regulation may reflect multidimensional adaptive processes rather than purely physiological mechanisms [22]. In female university athletes—who are already vulnerable to inadequate energy intake—this phenomenon may contribute to chronic energy deficiency if left unrecognized. From a practical standpoint, monitoring subjective sleep quality and gastrointestinal symptoms may provide a simple screening approach for identifying athletes at risk of insufficient post-exercise energy intake. Integrating sleep hygiene strategies and gastrointestinal health considerations into nutritional support programs may enhance recovery and reduce the risk of low energy availability.

This study has several limitations. First, the sample size was relatively small, and participants were recruited from a single university, which limits generalizability and may have reduced statistical power. Because multiple lifestyle variables were examined in this exploratory analysis, the possibility of type I error due to multiple comparisons cannot be excluded. In addition, the wide confidence intervals observed for several odds ratio estimates reflect limited statistical precision associated with the small sample size and should therefore be interpreted with caution. Second, all variables were assessed using self-reported questionnaires, and several constructs (e.g., sleep quality, stress, gastrointestinal symptoms, fatigue, and appetite loss) were measured using single-item responses. This approach may be subject to recall bias and limited construct validity. Third, objective measurements of sleep parameters, energy intake, hormonal markers, or training load were not available in the present study. These physiological and behavioral factors may influence both recovery status and appetite responses in athletes. Fourth, the cross-sectional design precludes causal inference; therefore, the directionality of the association between sleep quality, gastrointestinal symptoms, and post-exercise appetite loss cannot be determined. Finally, residual confounding by unmeasured variables such as training intensity, menstrual status, or nutritional knowledge cannot be excluded. Future studies with larger samples and longitudinal designs incorporating objective physiological measurements are needed to clarify causal pathways and validate these findings.

Taken together, the present findings provide preliminary insight into the interaction between lifestyle-related recovery conditions and appetite regulation in university athletes and should be regarded as hypothesis-generating, warranting confirmation in larger prospective studies.

## 5. Conclusions

This exploratory study demonstrates inter-individual variability in self-reported post-exercise appetite loss among female university athletes. The findings suggest that appetite responses following exercise are not explained solely by acute physiological mechanisms but are influenced by broader lifestyle-related factors. In particular, subjective sleep quality emerged as a key factor associated with post-exercise appetite loss in this population. Given the dual demands faced by university athletes, accumulated lifestyle strain and recovery imbalance may be associated with post-exercise nutritional behavior. Monitoring subjective sleep quality may therefore provide useful insight into recovery-related conditions in athletes who experience appetite loss after exercise. Future longitudinal and interventional studies are needed to further clarify the relationships between sleep, recovery, and appetite responses in athletes.

## Figures and Tables

**Table 1:** Prevalence of Post-Exercise Appetite Loss among Female University Athletes by Sports Discipline.

Sports Discipline	Appetite Loss *n* (%)	
	Present	Absent
All participants ^(1)^	26 (74.3)	9 (25.7)
Basketball (*n* = 12) ^(2)^	9 (75.0)	3 (25.0)
Soccer (*n* = 12) ^(2)^	9 (75.0)	3 (25.0)
Speed skating (*n* = 11) ^(2)^	8 (72.7)	3 (27.3)

Chi-square test, *p* = 0.881. ^(1)^ Percentages were calculated within each row based on the total sample. ^(2)^ Percentages were calculated within each row, based on each sports discipline.

**Table 2:** Bivariate Associations between Post-Exercise Appetite Loss and Lifestyle- and Health-Related Factors (*n* = 35).

Variable	Category	Appetite Loss *n* (%)		*p*-Value ^1^	φ	OR (95% CI) ^2^
		Present	Absent			
Subjective Sleep Quality	Good (*n* = 13)	4 (30.1)	9 (69.9)	0.006	0.50	11.63 (1.90–73.64)
	Fair/Poor (*n* = 22)	23 (90.9)	3 (9.1)			
Usual Appetite Loss	No (*n* = 20)	9 (60.0)	6 (40.0)	0.119	0.29	3.77 (0.76–18.73)
	Yes (*n* = 15)	17 (85.0)	3 (15.0)			
Diarrhea	No (*n* = 12)	11 (39.1)	9 (60.9)	0.015	0.43	25.61 (1.33–492.9) ^3^
	Yes (*n* = 23)	15 (100.0)	0 (0.0)			
Fatigue	No (*n* = 11)	16 (66.6)	8 (33.4)	0.178	0.23	5.00 (0.53–47.1)
	Yes (*n* = 24)	10 (90.9)	1 (9.1)			

Post-exercise appetite loss was dichotomized as “present” (“often” or “sometimes”) and “absent” (“never”). Subjective sleep quality, usual appetite loss, frequency of diarrhea, and frequency of fatigue were recategorized into binary variables to ensure statistical stability. ^1^ Fisher’s exact test (two-sided). ^2^ OR calculated as odds of appetite loss in athletes with diarrhea relative to those without diarrhea. ^3^ OR estimated using the Haldane–Anscombe correction (adding 0.5 to each cell) due to zero-cell counts; interpret with caution.

**Table 3:** Multivariable Logistic Regression Analysis for Post-Exercise Appetite Loss.

Variable	OR	95% CI	*p* Value
Sleep quality (fair/poor vs. good)	9.68	1.51–61.93	0.016
Usual appetite loss (yes vs. no)	2.44	0.40–14.79	0.333

An OR > 1 indicates a higher likelihood of appetite loss. Model fit: Hosmer–Lemeshow χ^2^ = 2.33, df = 1, *p* = 0.127. Discrimination: AUC = 0.79.

## Data Availability

The data presented in this study are available on reasonable request from the corresponding author. The data are not publicly available due to ethical restrictions.

## Supplementary Materials

The following supporting information can be downloaded at https://www.mdpi.com/article/10.3390/sports14040157/s1, Table S1: Questionnaire items used in the survey assessing appetite, sleep, stress, and health-related factors; Table S2: Additional statistical analyses examining associations between post-exercise appetite loss and lifestyle-, stress-, and symptom-related variables.

## Abbreviations

The following abbreviations are used in this manuscript:

CI	Confidence interval
OR	Odds ratio
