# Supplementary material for: Subjective Sleep Quality Is Associated with Post-Exercise Appetite Loss in Female University Athletes: An Exploratory Cross-Sectional Study
Source: Sports (Basel). 2026 Apr 16;14(4):157. doi: 10.3390/sports14040157 (PMC13120320; doi:10.3390/sports14040157)
Supplement: Supplementary file 1 [file sports-14-00157-s001.zip › sports-4177344_Supplementary_Table S1.pdf]

Supplementary Table S1. Questionnaire Items Assessing Appetite, Sleep, Stress, and Health-Related Factors.

The following questions ask about your lifestyle and health status over the past three months. Please answer each question based on your experiences during this period.

Appetite

1. **Post-exercise appetite loss**
   Have you experienced a decrease in appetite after exercise?
   - Often
   - Sometimes
   - Never
2. **Usual appetite loss**
   Have you experienced a decrease in appetite in daily life?
   - Often
   - Sometimes
   - Never

Sleep

3. **Sleep duration**
   What was your average sleep duration per night?
   - Less than 3 h
   - 3–4 h
   - 5–6 h
   - 7–8 h
   - 9 h or more
4. **Subjective sleep quality**
   How would you rate your overall sleep quality?
   - Good
   - Fair
   - Poor

Alcohol consumption and smoking

5. **Alcohol consumption**
   How often did you consume alcohol per week?
   - Every day
   - 5–6 times per week
   - 3–4 times per week
   - 1–2 times per week
   - Rarely or never
6. **Smoking status**
   What is your smoking status?
   - Current smoker
   - Former smoker
   - Never smoked

Dietary habits

7. **Meal frequency**
   What was your average number of meals per day?
   - One meal
   - Two meals
   - Three meals
   - More than three meals

8. **Snacking before exercise**
   Did you consume snacks before exercise?
   - Always
   - Sometimes
   - Never
9. **Usual meal quantity**
   How would you describe your usual meal quantity compared with others?
   - Enough
   - Moderate
   - Small

Stress

10. **Perceived stress level**
    How much stress did you experience?
    - Very high
    - High
    - Low
    - None
11. **Perceived stress tolerance**
    How would you rate your tolerance to stress?
    - Very high
    - Moderate
    - Low
    - Very low

Physical condition

12. **Stomachache or abdominal pain**
    How often did you experience stomachache or abdominal pain?
    - Often
    - Sometimes
    - Never
13. **Diarrhea**
    How often did you experience diarrhea?
    - Often
    - Sometimes
    - Never
14. **Headache**
    How often did you experience headaches?
    - Often
    - Sometimes
    - Never
15. **Fatigue**
    How often did you experience fatigue?
    - Often
    - Sometimes
    - Never
