# Supplementary material for: Subjective Sleep Quality Is Associated with Post-Exercise Appetite Loss in Female University Athletes: An Exploratory Cross-Sectional Study
Source: Sports (Basel). 2026 Apr 16;14(4):157. doi: 10.3390/sports14040157 (PMC13120320; doi:10.3390/sports14040157)
Supplement: Supplementary file 1 [file sports-14-00157-s001.zip › sports-4177344_Supplementary_Table_S2.pdf]

Supplementary Table S2. Associations between Post-Exercise Appetite Loss and Other Lifestyle-, Stress-, and Symptom-Related Variables (n = 35)

Variable	Category	Appetite Loss n (%)		p-value[1]	φ	OR (95% CI)[2]
		Present	Absent			
Sleep duration	≥7 h (n = 27)	20 (74.1)	7 (25.9)	1.000	−0.01	0.95 (0.15–5.86)
	<7 h (n = 8)	6 (75.0)	2 (25.0)			
Meal frequency	≥3/day (n = 3)	3 (100.0)	0 (0.0)	0.553	0.18	2.83 (0.13–60.21)[3]
	<3/day (n = 32)	23 (71.9)	9 (28.1)			
Snacking before exercise	Yes (n = 30)	22 (73.3)	8 (26.7)	1.000	−0.05	0.69 (0.07–7.11)
	No (n = 5)	4 (80.0)	1 (20.0)			
Usual meal quantity	Sufficient (n = 33)	24 (72.7)	9 (27.3)	1.000	−0.14	0.52 (0.02–11.77)[3]
	Insufficient (n = 2)	2 (100.0)	0 (0.0)			
Perceived stress level	High (n = 27)	21 (77.8)	6 (22.2)	0.396	0.15	2.10 (0.39–11.43)
	Low (n = 8)	5 (62.5)	3 (37.5)			
Stress tolerance	High (n = 24)	18 (75.0)	6 (25.0)	1.000	0.02	1.13 (0.22–5.67)
	Low (n = 11)	8 (72.7)	3 (27.3)			
Stomachache	Yes (n = 4)	4 (100.0)	0 (0.0)	0.553	0.21	3.80 (0.19–77.76)[3]
	No (n = 31)	22 (71.0)	9 (29.0)			
Headache	Yes (n = 9)	7 (77.8)	2 (22.2)	1.000	0.05	1.29 (0.21–7.76)
	No (n = 26)	19 (73.1)	7 (26.9)			

Post-exercise appetite loss was dichotomized as "present" ("often" or "sometimes") and "absent" ("never"). Predefined lifestyle-, stress-, and health-related variables were recategorized into binary variables to ensure statistical stability.

[1] Two-sided Fisher's exact test.

[2] Odds ratios (ORs) were calculated from 2×2 contingency tables.

[3] OR estimated using the Haldane–Anscombe correction due to zero-cell
